# Monitoring radon concentration in roman catacombs: a long-term analysis across different geological settings

**DOI:** 10.1007/s10653-025-02661-z

**Published:** 2025-07-25

**Authors:** Gaia Soldati, Maria Grazia Ciaccio, Gianfranco Galli, Valentina Cannelli, Antonio Piersanti

**Affiliations:** Istituto Nazionale di Geofisica e Vulcanologia, Via di Vigna Murata 605, 00143 Rome, Italy

**Keywords:** Indoor radon, Catacombs, Active monitoring, Radon risk assessment

## Abstract

Radon concentration monitoring was conducted in three Roman catacombs characterized by distinct geological settings, motivated by the anticipated high radon levels in indoor underground spaces—some of them carved into volcanic deposits—and the associated potential health risks for workers and visitors. The study was also inspired by the interest in an environment that is generally difficult to access and consequently little studied. Radon studies in catacombs existing in literature primarily focus on average radon levels; in this study, active monitoring instruments were employed to analyze radon concentrations both spatially and temporally across multiple time scales, enabling the detection of the gas’s full dynamics, including diurnal and seasonal variations as well as long-term trends. The extreme conditions of the catacombs, including high humidity, and elevated radon levels, posed significant challenges for accurate and continuous measurements. A considerable part of our work was devoted to finding the optimal configuration and installation of the instruments to mitigate environmental interference and operational issues and to ensure the recording of long and reliable time series of radon concentration. The results revealed notable differences in radon levels across the three catacombs, with clear correlations to the geological characteristics of each site. Temporal analyses indicated significant seasonal patterns, likely driven by temperature changes between summer and winter periods. Short-term fluctuations were also observed at some sites, providing insights into the dynamic behavior of radon influenced by environmental conditions. The study expands our understanding of radon dynamics in catacombs, with practical implications for the protection of individuals who frequently access such sites and for the development of effective radon mitigation strategies in underground cultural heritage environments. In addition, it highlights the importance of devising proper methodological adjustments when monitoring radon in such unique underground environments.

**Supplementary Information:**

The online version contains supplementary material available at 10.1007/s10653-025-02661-z.

## Introduction

The Roman catacombs, famous for being burial sites during the early centuries of Christianity, were essentially underground necropolises, and for a long period, they housed the remains of the deceased, in a context that reflected religious and cultural beliefs. At the time when the Christian faith was persecuted, the catacombs provided a secure place where Christians could gather to pray, celebrate religious rites, and bury their dead without fear of repression. The underground nature of the catacombs offered a certain level of discretion and protection from external threats. Today it is known that the risks that can make these environments dangerous also come from within, for instance the exposure to radon, a naturally occurring radioactive gas commonly found underground, that may accumulate at unsafe levels when trapped in enclosed spaces with inadequate ventilation, such as catacombs. Radon is known to be a Group A carcinogen (WHO, 2009), and it is the second leading cause of lung cancer after tobacco smoking. Although the Roman catacombs are no longer actively used as burial sites, it is important to consider the dangers related to the environment, that’s why it is so important an accurate monitoring of radon level and fluctuations to plan safety measures to protect the health of those who visit or work in these places.

Another key factor that motivated the decision to conduct radon measurements inside the catacombs is the uniqueness of this environment—not only in terms of its geological and historical characteristics but also due to the limited accessibility of such spaces. This challenge is reflected in the scarce number of scientific studies on this subject available in the literature. Among these, one can mention those of (Abdelzaher, 2011; Abd-Elzaher, 2014; Hafez et al., 1997), who repeatedly measured the gas concentration in the catacomb of Kom EI-Shuqafa, Alexandria, Egypt, considered one of the seven wonders of the Middle Ages. Using passive radon detectors, they could only obtain averaged values of radon activity concentrations, with monthly radon activity ranging from a few dozen to a few hundred Bq/m^3^, and seasonal variation peaking in summer and dropping in winter. This is the opposite of what is commonly observed in residential radon measurements, inside homes or buildings in general (Soldati et al., 2024, 2022b, 2022a). Quarto et al. (2014) inspected the Neapolitan catacombs: the catacombs of San Gennaro (second century AD, 5800 m^2^—the largest in Italy—with over 2000 loculi) and the catacombs of San Gaudioso (Paleochristian era, fourth-fifth century, the second largest in Naples). LR115 passive alpha detectors were exposed for two quarters, finding radon concentrations lower in winter than in summer; and diurnal variations were evaluated from continuous measurements by a Radim 5 monitor over a period of 10 days.

Other works deal with indoor radon in another type of underground or semi-underground spaces, often characterized by darkness, humidity, and an enclosed atmosphere, which share with the catacombs the function of burial sites: pyramids, crypts and tombs. The papers by Bigu et al. (2000) and Kenawy (1991) are focused on the Sakhm Khat Pyramid at Saggara and the great pyramid of Cheops in Giza, respectively, finding particularly dangerous radon levels (higher than 5000 Bq/m^3^) in the first site. Espinosa et al. (1997) shifted the focus to Mexico, where they measured average radon levels below 200 Bq/m^3^ into the pyramid of the sun at Teotihuacan. Cataldo et al. (2008) tracked the gas inside the crypt of the cathedral of Otranto, using E-PERM passive electrets and observing monthly levels of radon concentration, steadily over 300 Bq/m^3^. Finally, Salama et al. (2018) inspected three Egyptian tombs in the Saqqara region using both passive and active detectors, deployed for just 70 h, revealing that radon levels in summer are at least twice as high as those in winter.

What fundamentally sets this experiment apart from most of the indoor radon studies, including all those mentioned above that focus on such unique, isolated, and underground environments, is the use of active sensors deployed for a significantly long period of time inside a catacomb. Thanks to an agreement with the Pontifical Commission for Sacred Art (PCAS), we had the opportunity to access three Roman catacombs for a continuous two-year monitoring period. The importance of active radon monitoring for such an extended period lies primarily in the possibility to detect periodic patterns over a wide range of timescales, something unachievable with passive gas measurement methods typically used in environmental monitoring studies. Moreover, the availability of long-term data series is a fundamental prerequisite for enabling a comprehensive analysis of the relationship between radon emissions and both internal and external atmospheric conditions in the catacombs.

Indeed, meteorological conditions are known to be a key factor influencing radon migration, as rainfall, wind, and surface temperature gradients create pressure differences that may affect radon transport in porous media (Inan et al., 2012; Klusman & Webster, 1981). Although the role of meteorological factors in modulating soil radon emissions has been extensively studied over the past 50 years (Piersanti et al., 2015; Singh et al., 1988; Soldati et al., 2022a, 2024; Zmazek et al., 2003), their overall impact on radon observations remains difficult to consistently and routinely assess due to the complexity and site-specific nature of these interactions. Moreover, the relative significance of key variables such as surface temperature, precipitation, and pressure cannot be definitively established, with different studies yielding varying conclusions (Piersanti et al., 2015; Soldati et al., 2020; Zafrir et al., 2013).

In summary, given the need to address the issue of radon hazards within the catacombs—sites that are typically difficult to access and therefore understudied—we made use of radon detection instruments operating in active mode. These devices enabled us to acquire continuous measurements of gas concentration that lasted for as long as two full years. Compared to previous studies, typically relying on a very limited number of observations and on passive instruments that provide only average annual or seasonal gas concentration values, we could quantify absolute concentration levels and analyze temporal variations, including trends and periodicities across multiple time scales.

This made it possible for us to provide a qualitative interpretation of various factors contributing to this complex dynamic, many of which cannot be directly measured or controlled.

The article is structured as follows: the “Methods” section describes the geological framework and the characteristics of the monitored catacombs, along with the instruments employed and the challenges of obtaining continuous data in such a unique and harsh environment. The “Results and Discussion” section presents the time series of radon concentration, and the analyses performed to interpret the data and understand gas dynamics. Both periodic and non-periodic fluctuations at different timescales are discussed, as well as the influence of atmospheric parameters on radon emission from the ground. The final section summarizes the main findings and their implications, including potential health risks for visitors and workers in the catacombs and the impact on cultural heritage conservation.

## Materials and methods

### Geological setting

The outcropping geological units in the municipality of Rome, as well as those within the first 100 m of depth, along with the morphology of the Roman countryside, are the result of the geological history of the last 5 million years. During the Early Pliocene, post-orogenic sedimentation began in a subsiding environment linked to the expanding Apennine orogen-foredeep system. Marine sedimentation dates to the Early Pliocene, with a gradual decrease in sea depth until the Early Pleistocene, when a generalized uplift of the area occurred. From the Middle Pleistocene onward, depositional environments progressively became continental due to fluvial processes, strongly influenced by climatic and eustatic variations. Additionally, this period marks the beginning of volcanic activity in the Lazio region. Rome is situated in the distal zone of ignimbrite plateaus, where deposits from the Sabatini Volcanic District to the north interdigitate with those from the Colli Albani Volcano to the south. The Sabatini Volcanic District is characterized by a complex distribution of volcanic centers over time and space (Giordano, 2008). In Rome’s territory, distal facies of the largest ignimbritic deposits and fallout deposits are mainly found in the northern sector of the city, with smaller outcrops in the eastern part. The Colli Albani Volcano, on the other hand, is a complex central volcanic edifice that is currently quiescent. Throughout its evolution, it has undergone significant changes in eruption styles and rates. Its volcanic products, mainly tuffs, pyroclastic flows, and lava flows, have intermingled with volcanic deposits from the Sabatini area to the north.

The geological history of the Roman area determines the distribution of lithologies, even in its urbanized sectors. Within the Grande Raccordo Anulare (a major ring road encircling the capital), 62% of the deposits are volcanic, while the remainder consists of marine (0.5%), continental (12.6%), and Holocene (24.9%) deposits (Funiciello & Cologgi, 2008).

### The Roman catacombs

The widespread presence of volcanic deposits, particularly in the eastern area of Rome, has led to the formation of an extensive network of underground cavities. These intricate gallery systems, sometimes multi-leveled and reaching various depths, have occasionally caused subsidence phenomena, sometimes resulting in significant collapses. These man-made voids were excavated for different purposes, primarily for extracting construction materials such as pozzolanas and lithoid tuffs. Later, they were repurposed for religious needs, serving as various types of underground cemeteries, many of which reused abandoned quarries (Ventriglia, 1971 and references therein).

With the rise of Christianity, large and complex hypogeal burial sites were created to accommodate entire communities within a single necropolis. The ancient term for these monuments was “coemeterium”, derived from Greek, meaning “dormitory”. The term catacomb, now used for all Christian cemeteries, originates from the Roman toponym “catacumbas” (“near the cavities”), which, in the fourth century, referred to a site located at the third mile of the via Appia. In that area, from the third century, the extensive underground cemetery of St. Sebastian was excavated, and named “cymiterium catacumbas”.

The most important Christian catacombs in Italy are those of Rome, located outside the ancient city walls but still integrated into the extended urban fabric. There are over 60 catacombs, spanning multiple levels and extending for a total of 150–170 km of galleries. These sacred sites, of extraordinary beauty, stand as remarkable witnesses to a distant past.

**Fig. 1:**
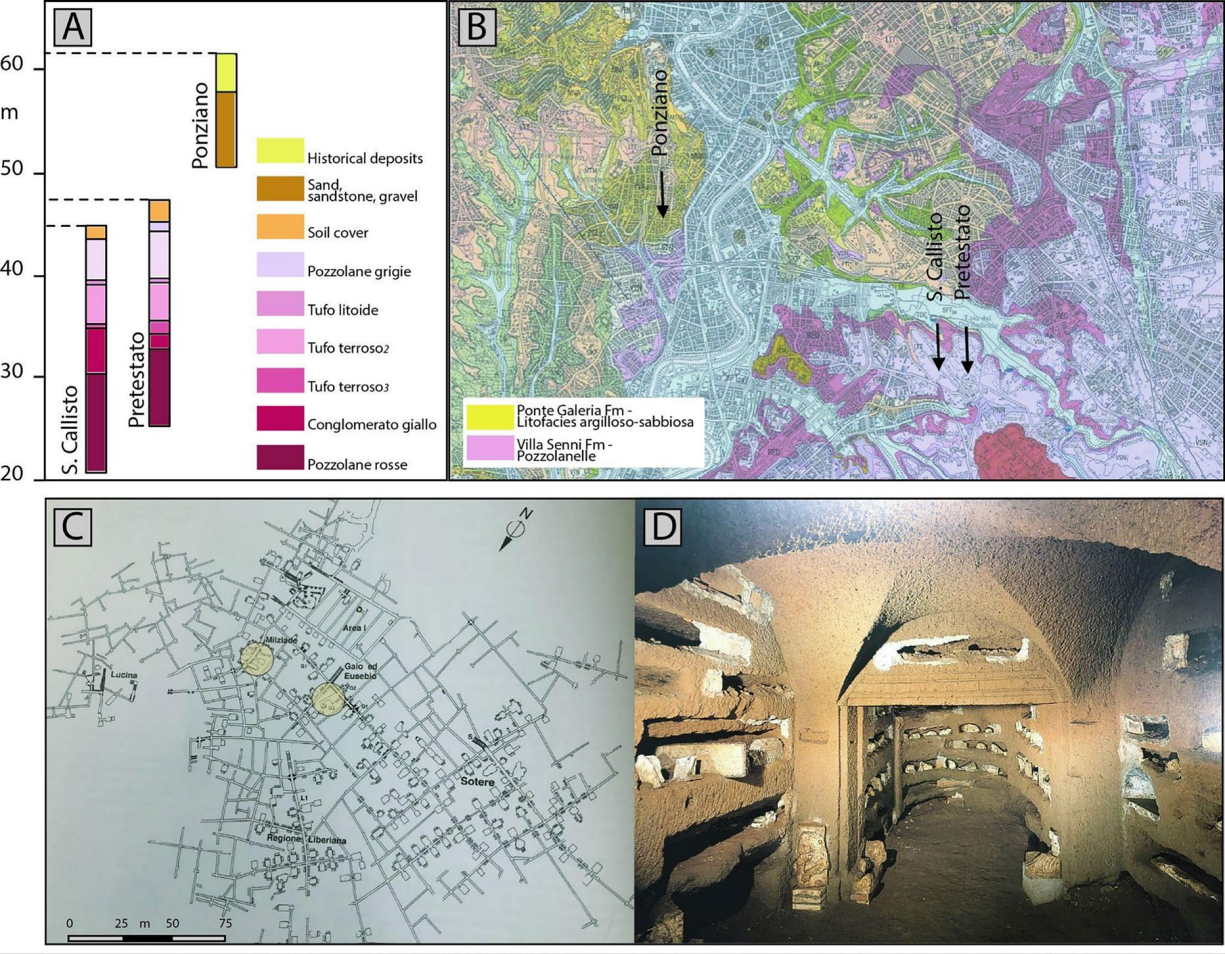
Stratigraphic relationship between sediments **A** in the studied catacombs (Ventriglia, 2002), and their location **B** on the geological map of the Municipality of Rome (Funiciello et al., 2008). Map of the San Callisto catacomb (C) with the location of the stations (yellow circles), and example of the interior of the catacomb (D), the “Cubicolo architettonico” in the Sotere region (Fiocchi Nicolai et al., 1999)

They are managed by Pontificia Commissione di Archeologia Sacra and are classified based on their geographic position in relation to the Tiber River: Trasteverina catacombs (to the right of the Tiber) and the Cistiberina catacombs (to the left) (Ventriglia, 2002). The three sites monitored in this experiment belong to both categories: one lies on the right of the Tiber (the catacomb of Ponziano) and two on the left (San Callisto and Pretestato), and they developed within different rock types. The location of these sites, superimposed on the geological map of the municipality of Rome is shown in Fig. 1, along with the stratigraphic relationship between sediments.

The catacombs of Ponziano (also known as the catacombs of Abdon and Sennen or Coemeterium ad Ursum Pileatum) are in the ‘Monteverde Vecchio’ district and remain closed to the public. The surface elevation is 62 m above sea level (m a.s.l.), while the lowest excavated level reaches approximately 49 m a.s.l. The underground structures lie within Pleistocene sandy and gravelly clays of the Ponte Galeria Formation, intersecting the local aquifer.

The catacombs of San Callisto, open to the public, are the most famous, largest, and best-preserved in Rome, being the oldest official cemetery of the Christian community. Their entrance is on Via Appia Antica. Built around the mid-second century, they housed over 500,000 Christian burials, including dozens of martyrs and 16 popes. Covering an area of 15 hectares, the catacombs extend nearly 20 km underground, descending to great depths across five levels. The galleries, flanked by loculi (carved burial niches), are arranged in two or three superimposed rows. The surface elevation is 45 m a.s.l., while the tunnels extend down approximately 24 m, cutting through different lithotypes of the Lazio pozzolanic complex, including Pozzolanella, Tufo Lionato, Tufo Terroso, Conglomerato Giallo, and Pozzolana Rossa. Among these, Tufo Lionato is the most widespread rock in the Roman area. It is an ignimbrite from the Colli Albani Volcano, part of the Villa Senni Eruptive Unit, with a radiometric age of approximately 338,000 years (De Casa, G. et al., 1999).

The catacombs of Pretestato, located west of San Callisto, lie within the same geological formations. The ground surface elevation ranges from 46 to 51 m a.s.l. Though not open to the public, this necropolis spans over 11,000 square meters.

A map of the tunnels of S.Callisto at level −1, where two instruments are positioned, is given in Fig. 1C, along with an illustrative photo (Fig. 1D) of the galleries, with stacked loculi.

### Instrumentation and experiment design

The two typologies of instruments employed in the experiment are the small sized solid-state commercial radon detectors Algade© AER-C and AirThings© Corentium Plus. AER-C by Algade© (http://www.algade.com/) provides measurements and local data storage for time, radon, temperature and relative humidity, Corentium Plus by AirThings© (https://airthings.com/) provides the same of AER-C, plus pressure. These instruments, tested for efficiency over a decade within the IRON network (Cannelli et al., 2018), were originally designed for consumer applications but demonstrated performance comparable to scientific-grade devices. To meet scientific standards, a customized correction function was developed for each sensor through a characterization procedure conducted at the INGV Radionuclide Laboratory (Galli et al., 2019). The correction formula R(s,RH,T), which accounts for the instrument response (s), relative humidity (RH), and temperature (T) readings, ensures data accuracy and reliability under all conditions. Notably, humidity influences the collection of short-lived alpha-emitting radon daughters on the solid-state detector.

The set-up of the instruments became definitive after a period of testing of four months, which considered the (unusual) environmental conditions. The catacombs, unlike a classic urban context, have almost constant temperature and humidity values, which are minimally affected by the season. A temperature difference ranging between 1 (Pretestato) and 5 degrees (San Callisto) was measured, with average values ​​between 15 and 20 degrees, and a very high relative humidity value (about 90–100%) in all the catacombs. Considering that both types of instruments are recommended to operate at a temperature range of 0–40 °C, and at relative humidity < 80%, Gewiss© boxes and silica gel (50 g) were employed to protect instruments from condensation. We have verified that this method allows to keep under control the level of humidity; however, even at humidity values close to 100%, the correct performance of the instruments has been tested and verified.

**Fig. 2:**
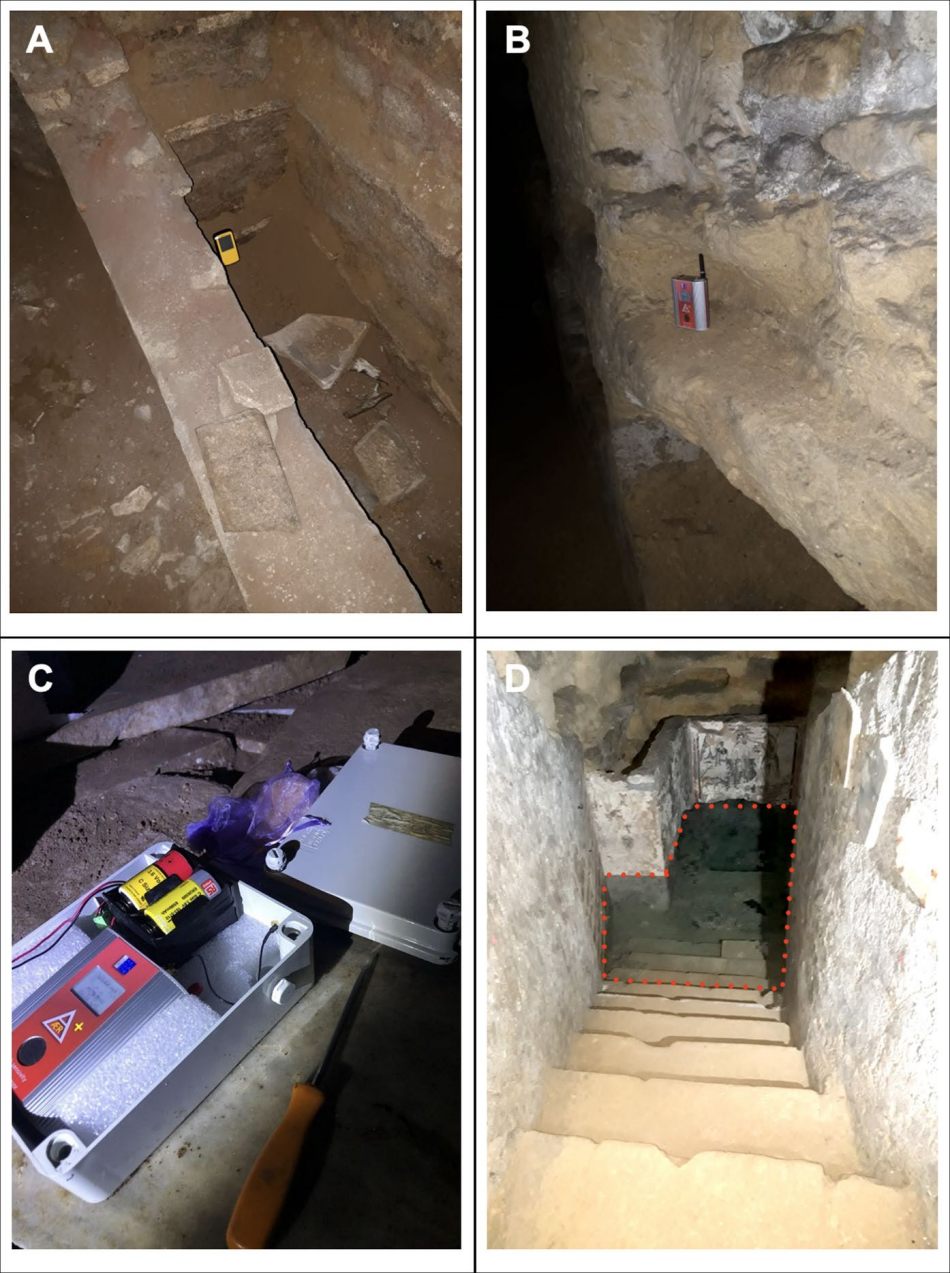
Corentium Plus instrument (**A**) and AER-C instrument (**B**) installed during the test period; Examples of AER-C instrument powered with a battery pack in a Gewiss box with Gore-Tex caps on the side (**C**); water pool (**D**) at the base of the Ponziano catacomb (the water level is indicated with a red dotted line)

A total of six instruments was installed, two for each catacomb (the station code corresponds to the location: “CALL” stands for S.Callisto, “PONZ” for Ponziano, “PRET” for Pretestato). Of these, one is enclosed in a sealed Gewiss© box with silica gel inside and the other in the same configuration but with Gore-Tex caps, which allow a few liters per min of air flow and, as a consequence, a greater internal diffusion of radon. The sampling time was set at one hour for all instruments. Examples of installation in the catacombs during the testing period are shown in Fig. 2A, B, while Fig. 2C displays an instrument with the current configuration (box with Gore-Tex caps).

It should be noted that the time series of radon concentration measured at the different stations are influenced by the location (for instance if they are far apart the entrance) and the depth of the instruments into the catacomb, as well as by the fact that among the monitored sites, only S. Callisto is open to the public.

In the context of the site characterization where the experiment was conducted, the catacomb of Ponziano stands out from the others because it intersects the groundwater; this makes it possible to measure also the concentration of radon dissolved in the water of a small pool at its base (Fig. 2D), at the depth of 10 m below the street level. The retrieved values, shown in supplementary Table 1, are quite low throughout the duration of the experiment and, for this reason, they have a large uncertainty.

## Results and discussion

### Radon data

**Fig. 3:**
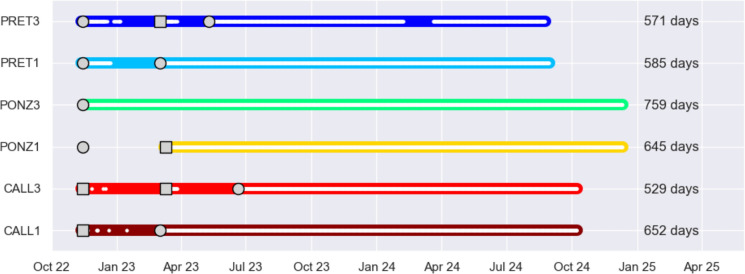
Colour lines indicate the recording intervals of the 6 stations, and the white dots represent the days where radon data were actually recorded, while their number is reported at the end of the intervals. Gray squares and circles correspond to the installation of a Corenthium-Airthing radon detector and an Algade-AER radon detector, respectively

Figure 3, displaying the recording intervals of the 6 stations, shows that despite the experiment started in November 2022, the data collected (white dots) are few and sparse during the first three months. This is due to the aggressive environmental conditions inside the catacombs, particularly the very high humidity combined with relatively elevated temperature, which degraded or even halted the functioning of some instruments until various attempts to protect them resulted in an optimal setup. Therefore, the time series were truncated before March 2023, keeping only the data recorded onwards. Even after this operation, there are still gaps in the CALL3 and PRET3 time series around April–May 2023, due to instrument damage and replacement, and in PRET3 in February–March 2024, caused by the battery running out prematurely. Up to now, although some data are still missing, the collected radon time series have length ranging from 529 days (CALL3) to 759 days (PONZ3), meaning that all stations provided recordings for at least 1.5 years. Working with a sampling rate of 1 h, we are left with a minimum of 11,000 radon data for each instrument.

Table 1 includes the main statistical values of the recorded radon time series, and shows that the highest radon levels, both in terms of average and maximum, are observed in the catacomb of Pretestato, followed by the catacomb of S. Callisto, and finally by that of Ponziano. This is consistent with the fact that the latter is not located on volcanic deposits.

**Table 1:** Descriptive statistics of the radon time series recorded since March 1, 2023 (mean, minimum and maximum values of radon concentration (Bq/m^3^), standard deviation, and total number of data on which they are computed)

	CALL1 Caps	CALL3 No Caps	PONZ1 No Caps	PONZ3 Caps	PRET1 No Caps	PRET3 Caps
Mean	16,754	1882	163	2157	8063	45,418
Min	59	0	0	45	15	0
Max	54,731	30,159	3764	14,365	102,474	107,738
Std	12,355	2796	349	1285	12,184	19,831
Count	14,036	11,853	15,464	15,646	12,972	11,002

For each station is indicated the setup type: box with Gore-Tex caps (“Caps”) or sealed box (“No Caps”)

As can be seen from the table, and from the following figures, in each of the catacombs, the two stations record significantly different radon values due to the distinct configuration; instruments placed inside a sealed box (CALL3, PRET1, PONZ1) are more isolated from the external environment and record lower values compared to those installed in boxes equipped with Gore-Tex caps (CALL1, PRET3, PONZ3), which allow greater internal radon diffusion. Both configurations provide scientifically significant data, but only the latter offers values that can be directly translated into radon concentrations useful for radiation protection purposes.

Note that the dispersion of radon values observed in stations with completely sealed Gewiss box is significantly greater than that observed in the stations with Goretex caps box, which however present overall higher concentration values ​​as the internal diffusion of radon is larger.

**Fig. 4:**
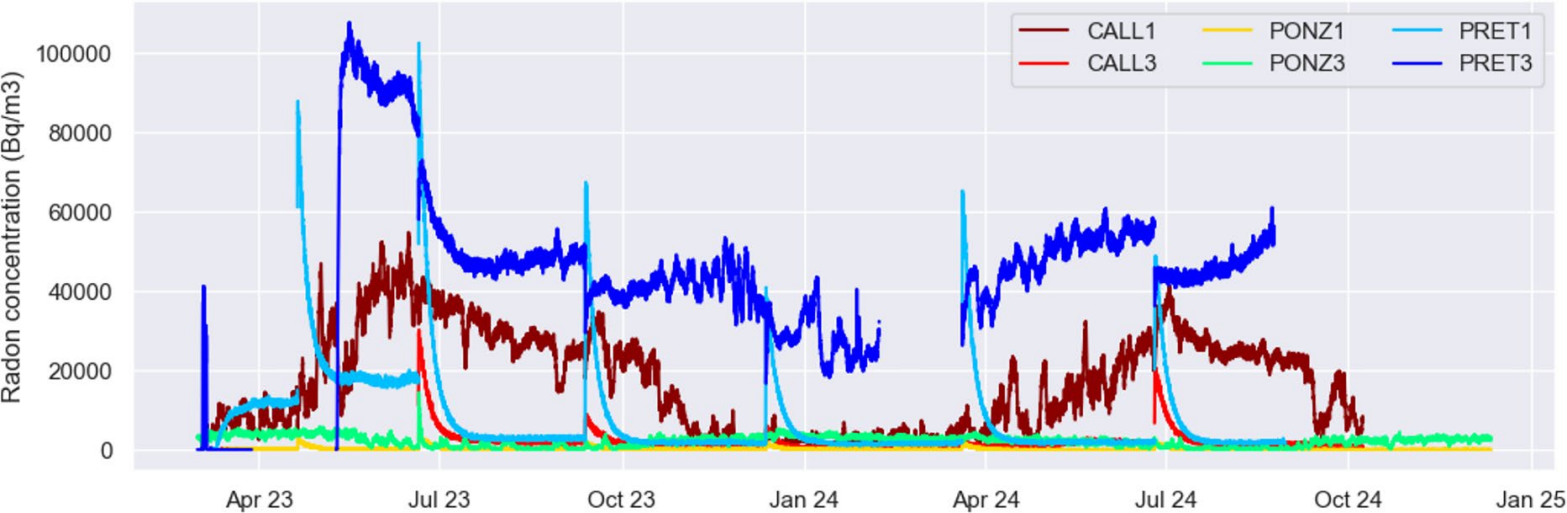
Radon concentration (Bq/m^3^) observed at the 6 stations

Figure 4 displays the temporal variations of radon concentration observed at the 6 stations, showing extremely high levels at the catacombs of Pretestato (blue), where radon values exceed 100,000 Bq/m^3^, and at S. Callisto (dark red), with maximum values around 50,000 Bq/m^3^. Such elevated concentration of gas is explained by the fact that both catacombs are carved into the tuff that originated from the eruptions of the Alban Hills volcanic system, characterised by significant content of Uranium, from whose decay Radon (Rn-222) is produced. In comparison, the radon time series recorded inside the catacomb of Ponziano (green) reach more limited values.

**Fig. 5:**
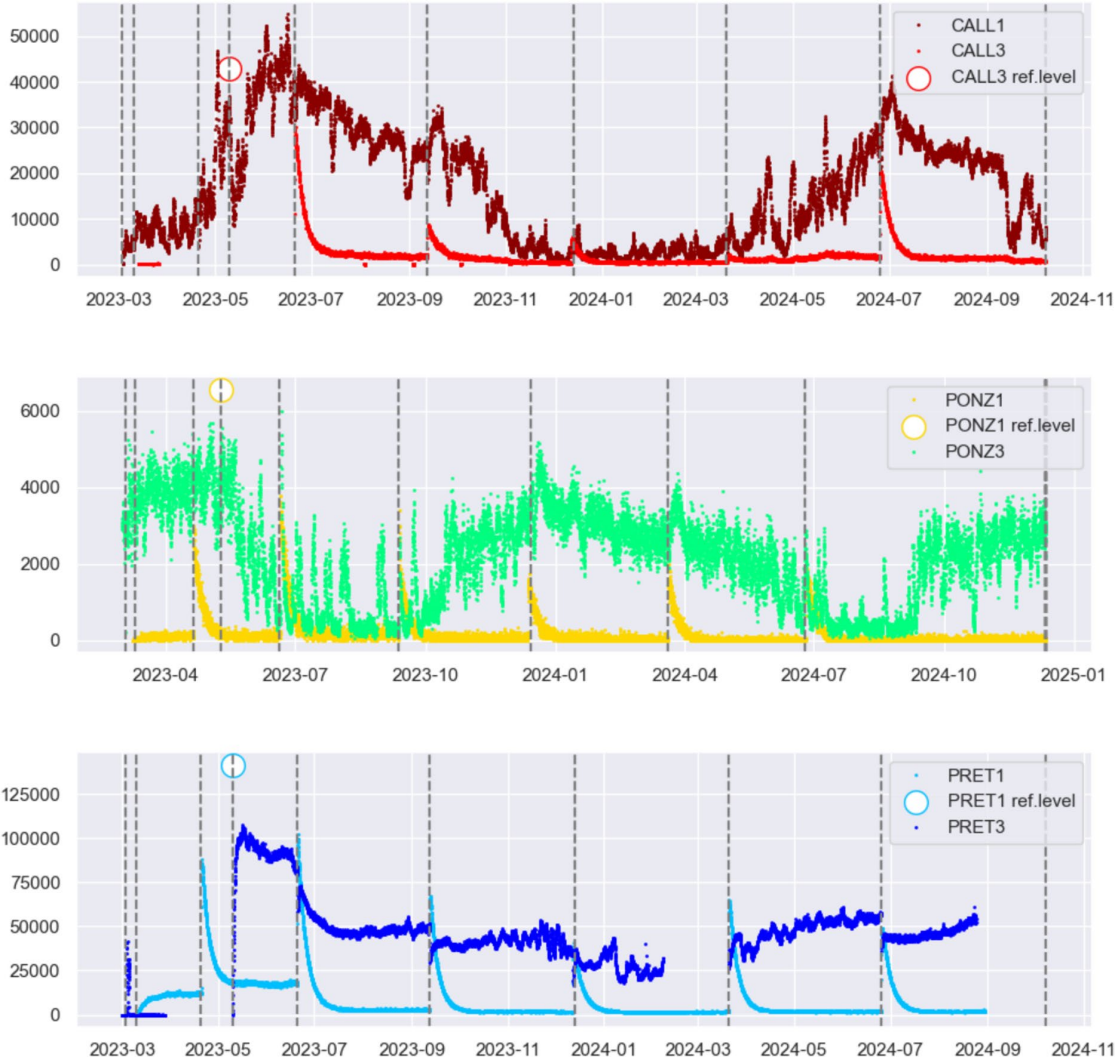
Radon concentration (Bq/m^3^) plotted separately for each catacomb. White circle refers to the value of radon concentration measured by an instrument without a box, positioned close to the one in a closed box for a few hours. Dashed lines refer to the days when maintenance activities were performed at the stations, such as downloading data or replacing batteries

Plotting the radon time series separately for each catacomb (Fig. 5), what immediately stands out is the difference in the curves recorded by stations with Gore-Tex caps (dark red, green, blue), which appear continuous almost everywhere, compared to those from stations in sealed boxes (light red, yellow, light blue). The latter show spikes on days corresponding to maintenance or data retrieval activities (marked by dashed vertical lines), as opening the box exposes the instrument to radon levels significantly higher than those diffusing inside the closed box.

The data from instruments in boxes with Gore-Tex caps and the maximum radon values recorded upon opening the sealed boxes during data retrieval are both indicative, albeit as lower limits, of the actual radon concentrations in the measurement environments. The actual values of radon concentration in the three catacombs were measured only once, on May 10, 2023, by instruments without protective casing, which were placed for a few hours next to those in sealed casings; the radon levels recorded are plotted as white circles in Fig. 5.

### Radon gas dynamics

#### Radon seasonal periodicity

**Fig. 6:**
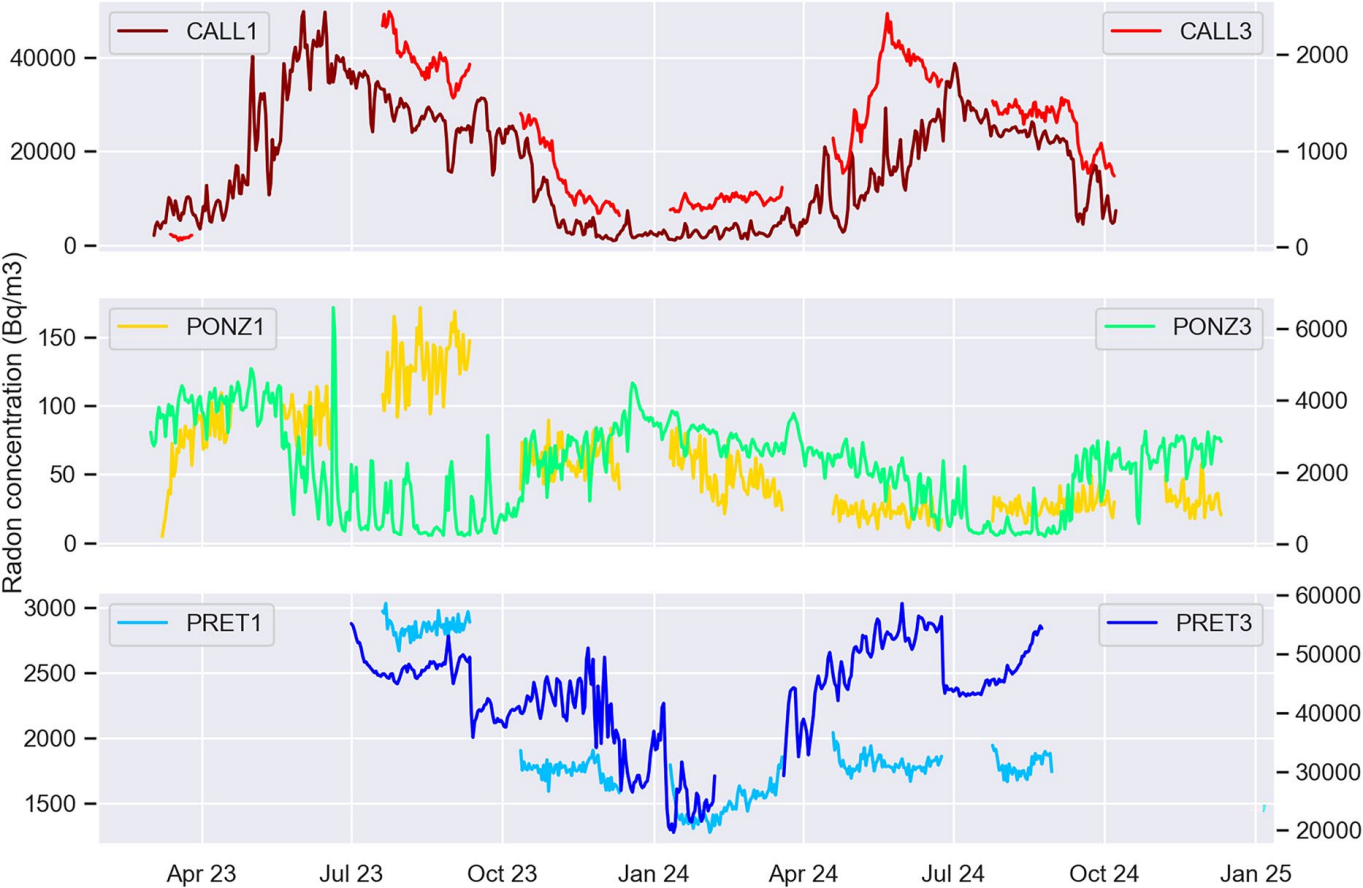
Daily average of radon concentration (Bq/m^3^) recorded in each catacomb by the two stations installed there

To highlight the temporal variations in radon concentration and compare the trends and periodicity of the series recorded by the stations with sealed casings to those enclosed with Gore-Tex caps, the 30 days of data (corresponding to 7–8 times the half-life of ^222^Rn) following the opening of the sealed casings, because they show large spikes which mask the global trend of temporal radon variations. The resulting time series are shown in Fig. 6, where each panel refers to a single catacomb. The radon peak observed in June 2023 at PONZ3, and due to the opening of the box containing the station after temporarily transporting it to a deeper level in the catacomb, is quickly recovered due to the greater air circulation inside the box ensured by the caps.

Focusing on the long term (yearly) periodicity, in the catacomb of S. Callisto, the radon series observed by instruments with a closed box has the same seasonality as the one with caps. The same holds true In Pretestato, although the similarity is discernable only after cutting the time series to neglect the highest radon values recorded in June 2023. In Ponziano catacomb, radon levels are very low, especially during the second year of the experiment; therefore, fluctuations at the two stations are visible (and seem to have opposite seasonality) just until the beginning of 2024. Radon observed at PONZ3 differs from PONZ1 and from the series observed in the other catacombs because it is located at street level, at a shallower depth compared to the other station, and closer to the entrance of the catacomb.

The reason why radon in catacombs or in other underground or enclosed spaces like caves, mines, galleries, and even pyramides, tends to reach maximum values during the hottest months of the year depends on the fact that temperatures are often constant, so that these spaces tend to be cooler than the outside during the summer and warmer during the winter. The difference between external and underground temperature generates a gradient of air density capable of inducing an airflow from the exterior to the interior during the cold season (thus diluting the underground radon gas and reducing its concentration), this migration is prevented during the warm season resulting in an increase of underground radon concentration.

**Fig. 7:**
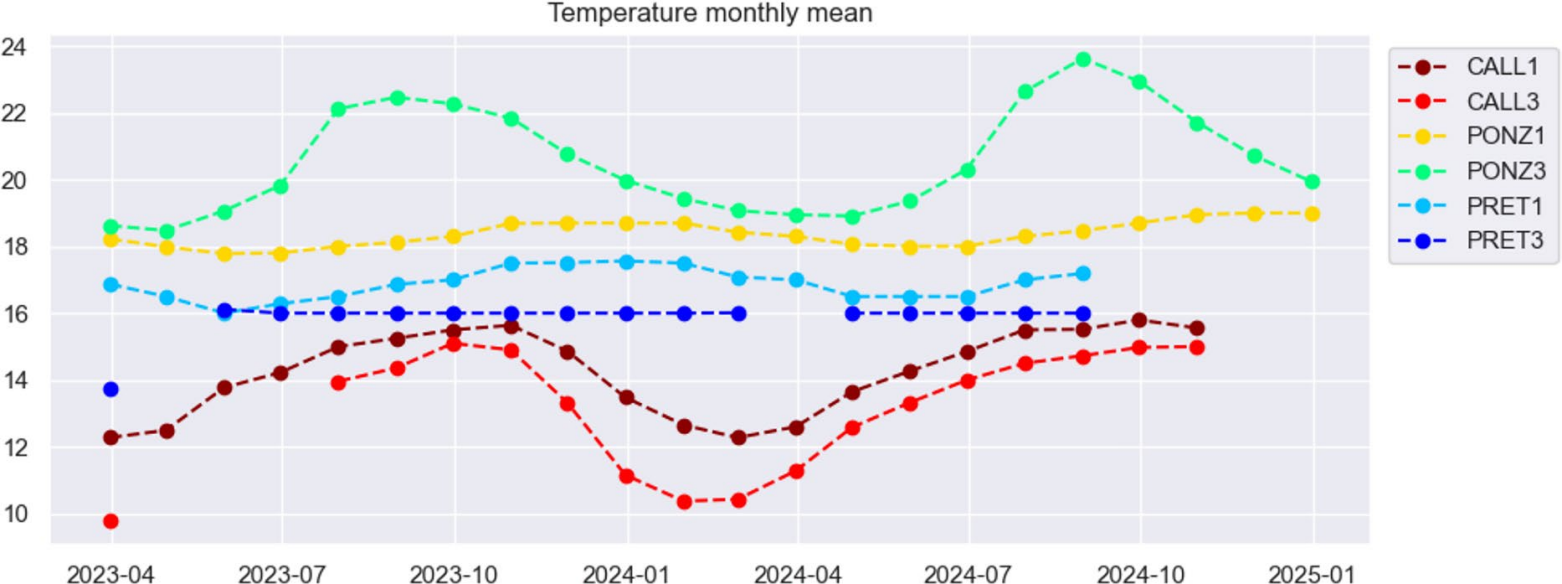
Monthly average of internal temperature (°C) recorded at each station

In the following, the relationship between radon and external temperature will be investigated in more detail, while here radon seasonal fluctuations are compared to those of internal temperature, recorded by the same instruments capable of detecting radon concentration, and shown in Fig. 7. Where seasonal temperature variations are more pronounced, radon also exhibits a rather distinct periodicity on that timescale. In contrast, at locations where the temperature remains essentially constant throughout the year, it is more challenging to discern seasonality in the radon series. This observation highlights the influence of temperature on gas emissions in an environment as unique and isolated as the catacombs (Przylibski, 1999; Zafrir et al., 2013).

The differences in temperature fluctuations recorded by the two instruments at the Ponziano site are due to their different depths and distances from the entrance of the catacomb. PONZ1 is located about 9 m below the surface, while PONZ3 is positioned at ground level and much closer to the entrance. Consequently, the seasonal pattern recorded at PONZ3 closely mirrors that of the outdoor air, with noticeably higher temperatures during the warmer months.

**Fig. 8:**
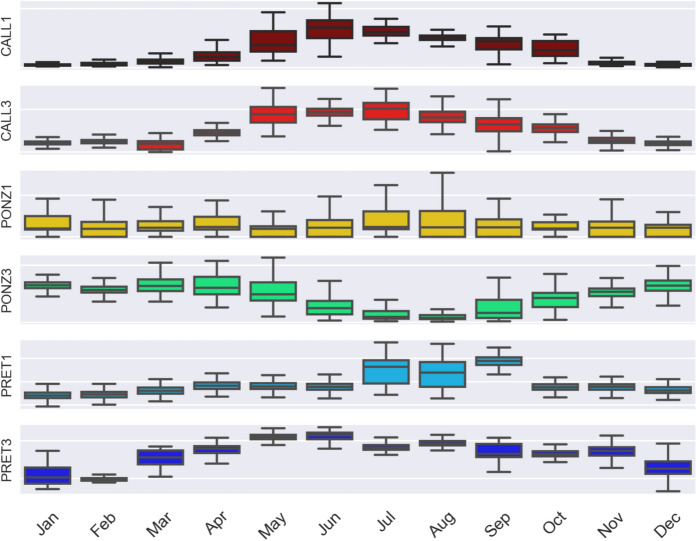
Boxplot (box-and-whisker plot) of radon concentration recorded at the stations

The box-and-whisker plots in Fig. 8 provide an alternative way to represent the distribution of the radon dataset and a summary of key data characteristics. They confirm the observations that the seasonality of the radon time series is clear and of the same sign at stations CALL1, CALL3, and partially PRET1, PRET3 (with potential outliers); of the opposite sign at PONZ3, and difficult to evaluate at PONZ1 (maybe because of the very low radon values detected, at the limit of instrument resolution), where radon concentration seems to vary with higher frequency (about three months).

**Fig. 9:**
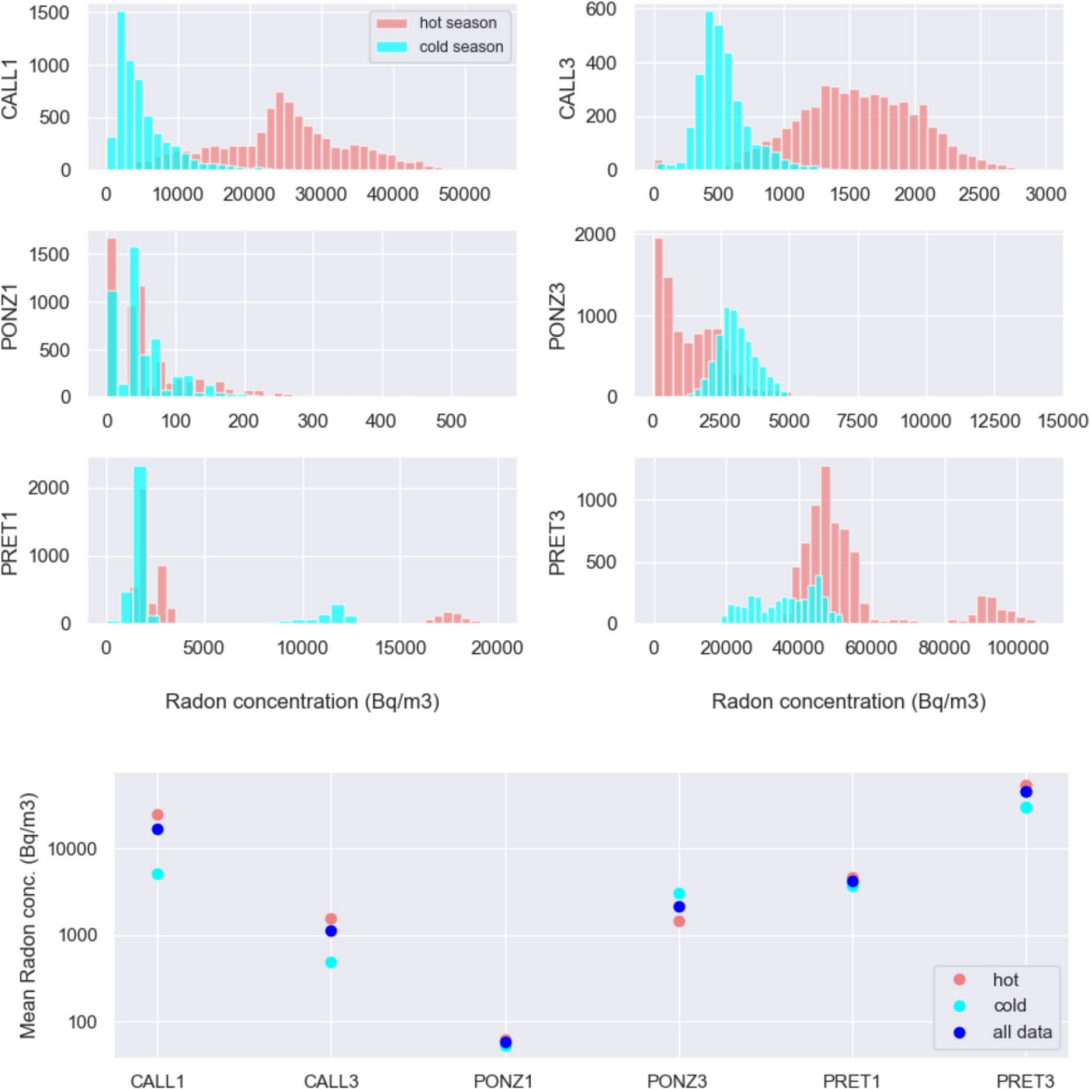
Top: Histogram of radon data frequencies. Coral/cyan colors correspond to the frequency of radon data recorded during the warmer season (May to October) and during the colder one (November to April). Bottom: Mean value of radon concentration recorded at the stations and computed from all the data, and from data recorded during the warm and the cold season (right)

Figure 9 (top panel) shows the distribution of frequencies of radon data measured in the interval May–October (hot season at the latitude of Rome) and November–April (cold season).

The histogram of data frequencies computed during the warm season is shifted towards higher values for the stations located at the catacombs of S.Callisto and Pretestato, and towards smaller values for station PONZ3. All these stations present a bimodal distribution of the whole data set, which confirms the difference in the summer–winter character of the radon data. Although even at PONZ1 the average radon value is higher in summer than in winter (see Fig. 9, bottom panel), the difference in the distribution of data collected by that station during warmer and colder months is much more subtle. Differentiating the data on the basis of summer/winter (taking the three hottest/coldest months) instead of by warm/cold season, the result does not change, but the discrepancy between radon values in the two intervals increases slightly.

### Radon daily periodicity

**Fig. 10:**
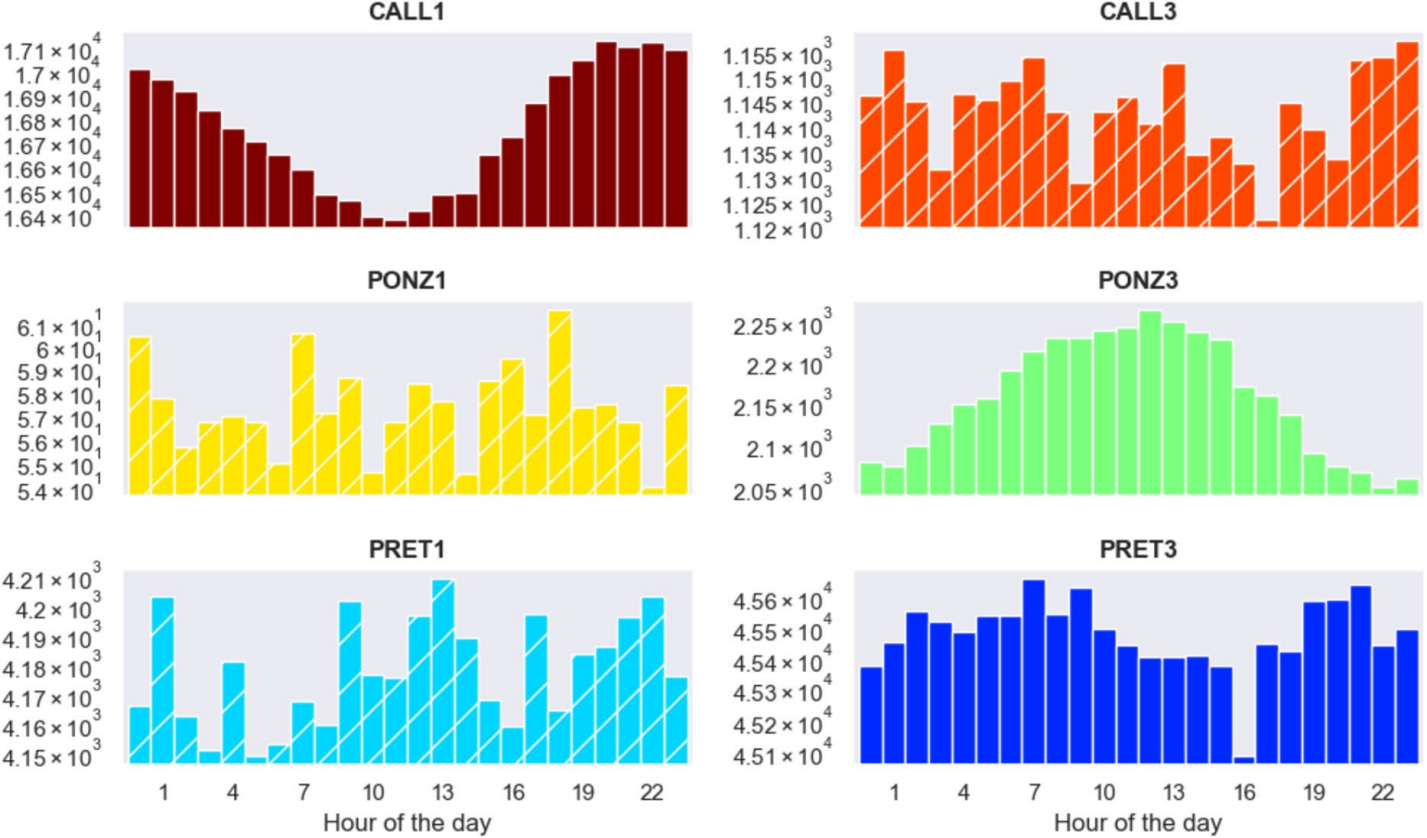
Average radon concentration (Bq/m^3^) as a function of hour. Solid color and hatched bars refer to instruments enclosed in boxes with and without Gore-Tex caps, respectively

Radon gas emissions can exhibit periodicity at different time scales due to various environmental, geological, and anthropogenic factors. Although seasonal periodicity is the most evident when analyzing long time series, the Fourier spectrum of radon concentration shows a significant peak at the frequency of 1 day, more pronounced at stations CALL1 and PONZ3. Figure 10 shows how radon concentration changes during the day: the stations with instruments in boxes with caps (solid colors) are capable of better catching the variability of radon concentrations on this time scale. At CALL1 radon levels appear to reach their minimum between 8 and 10 AM, right after the opening of the catacomb for ventilation before the visitors'entry, while at PONZ3, the minimum occurs between 8 PM and 2 AM; radon levels at station PRET3 are more variable, with an unexpectedly marked minimum at 4 PM.

Despite the daily variations of radon are very small—the logarithmic scale amplifies these fluctuations, but on a linear scale, the levels appear almost constant (see the table with percentage variations for'all data')—they are significant because the daily periodicity is perfectly outlined, at least for CALL1 (in a catacomb which is highly frequented by visitors) and PONZ3 (the stations in Ponziano catacomb closest to the surface, as indicated by the larger scale of radon level compared to PONZ1). Since the catacomb of Pretestato is rarely visited, recognizing this periodicity is more critical compared to S. Callisto. Radon time series measured by sensors in sealed boxes (hatched bars in Fig. 10) are noisier in terms of daily fluctuations; this is an effect of the sealed box, which acts as a low-pass filter, cuts radon concentration values increasing their uncertainties and prevents mapping small-scale time variations.

Even at hourly scale, radon fluctuations exhibit marked periodicity at the stations where temperature variations along the day are more evident. This suggests a direct correlation between short-term temperature changes and radon dynamics (Przylibski, 1999; Zafrir et al., 2013), as shifts in temperature can influence the pressure gradients and airflow patterns that drive radon dispersion. Such findings underscore the importance of considering even minor temperature variations when studying radon behavior, particularly in enclosed or semi-enclosed environments where air exchange is limited.

Examining the plot of radon time series month by month (not shown for the sake of brevity), radon exhibits daily periodicity during the cold months from March to May at CALL1 and during March-June at PONZ3. At the other stations, it is not possible to detect such fine periodicity. Many factors influence gas periodicity: the depth of measurement (close-to-surface measurements show stronger daily and seasonal effects, while deeper locations are more affected by geological processes), human activities (changes in ventilation changes or heating/cooling patterns may affect radon dispersion indoors) and type of installation (configuration and placement of sensors can affect their sensitivity and their ability to detect small-scale periodic variations).

### Radon trend

**Fig. 11:**
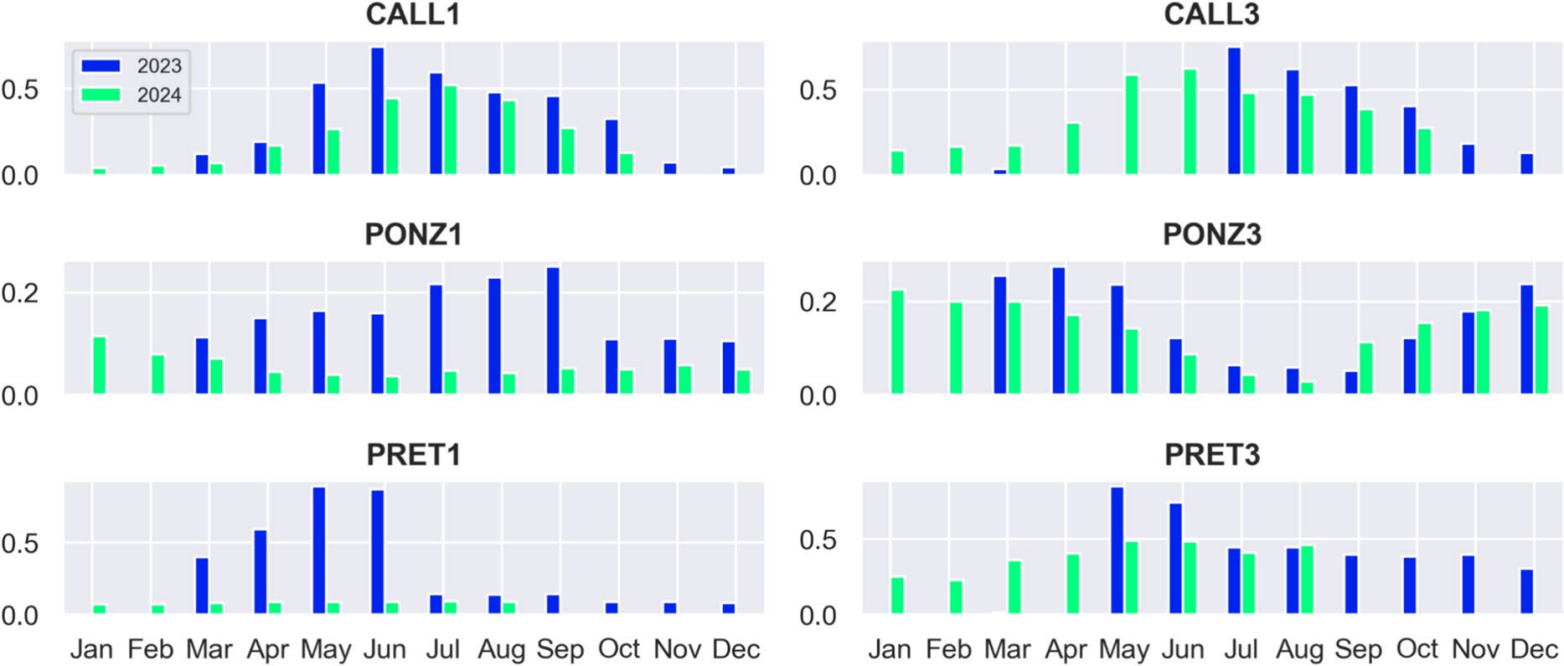
Monthly average of normalized radon concentration (Bq/m^3^) recorded by each station categorized by year

The availability of long time series spanning 1.5–2 years allows for the analysis of potential year-to-year trends. Figure 11 shows the monthly means of normalized radon concentration recorded by the stations over the two years of the experiment. The plots evidence a decrease of radon levels in 2024 compared to the same days/months in 2023.

**Fig. 12:**
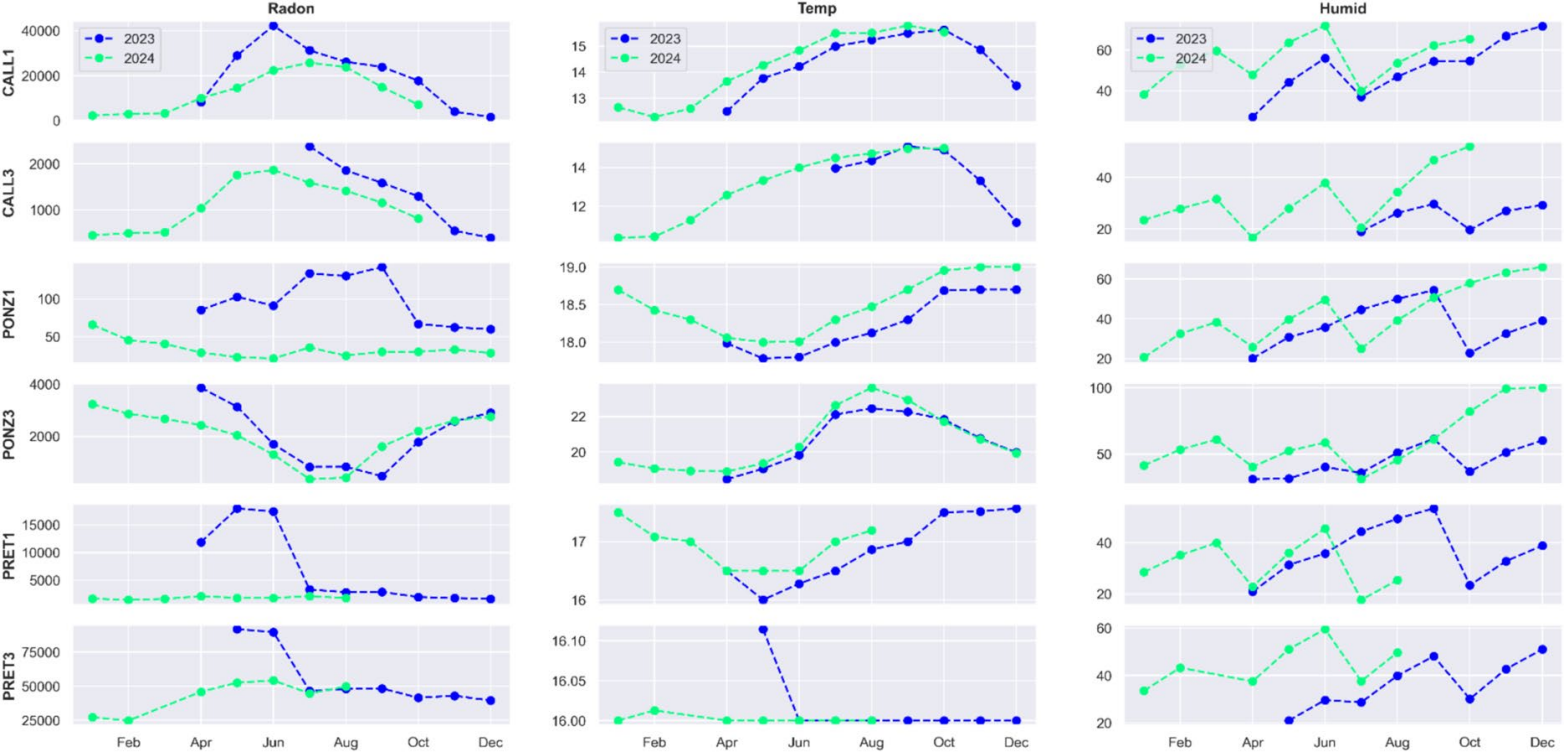
Monthly average of radon concentration (Bq/m^3^), temperature (°C) and relative humidity (%) recorded by each station categorized by year

The subsequent Fig. 12 reveals that the observed reduction in radon concentration is accompanied by a simultaneous increase in temperature and relative humidity. The relationship between radon and environmental parameters will be discussed in the next section.

### Radon and meteorological parameters

Over long periods, seasonal variations in air temperature predominantly influence radon anomalies, showing a correlation with sign which is strongly site dependent. The expected behaviour in underground or enclosed spaces like catacombs is positive correlation between the two time series, with maximum radon values recorded during the hottest months of the year instead of during the winter, as is commonly observed when indoor radon is measured in dwellings or workplaces (Soldati et al., 2022b, 2024). In Section “Radon Seasonal Periodicity”, this trend was confirmed in the catacomb of S.Callisto, while in Ponziano (PONZ3) long scale temporal changes of radon and temperature appear of opposite sign, and in Pretestato catacomb, the relationship radon-internal temperature is rather unclear (temperature is almost uniform throughout the seasons).

**Fig. 13:**
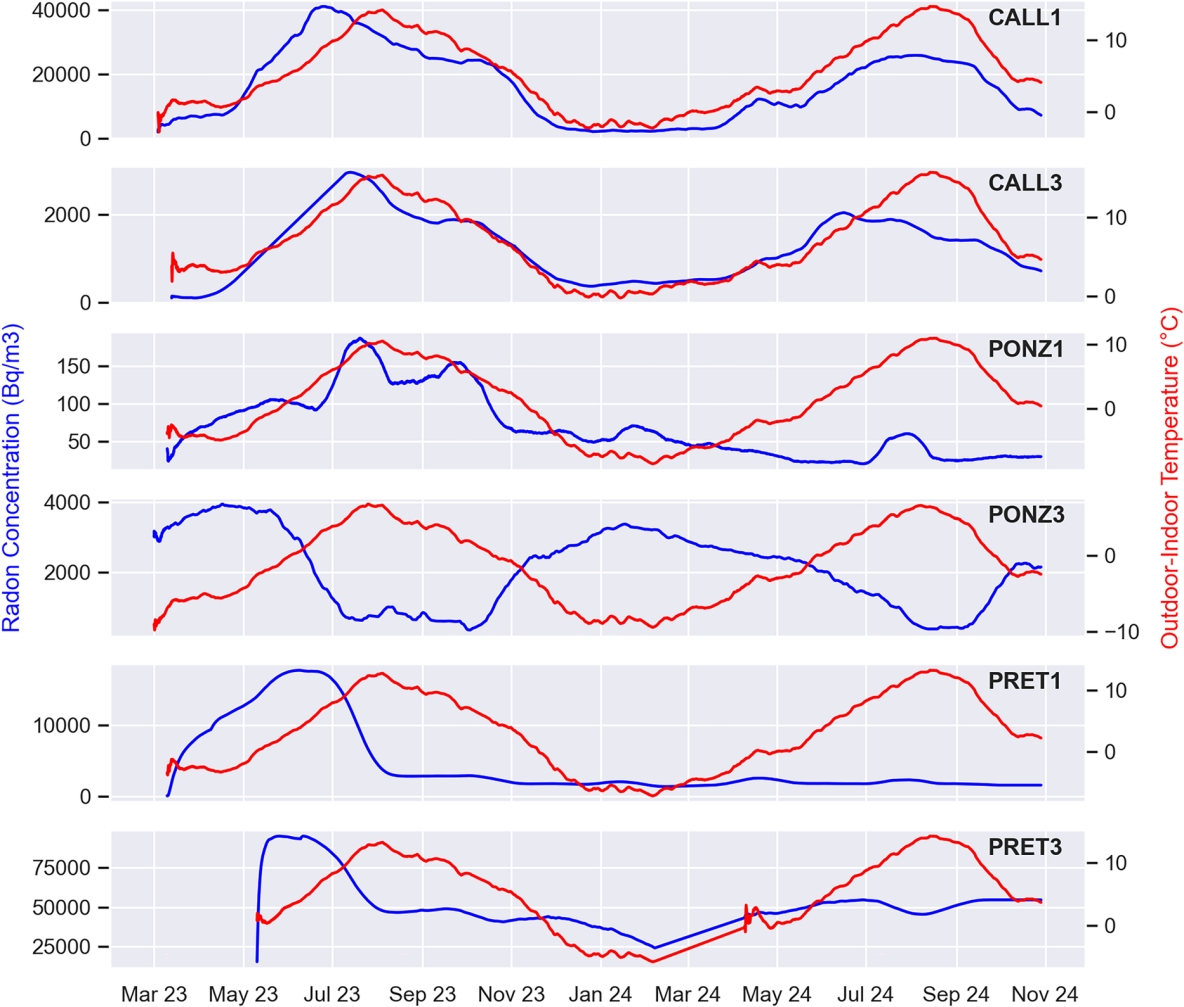
30-day moving means of radon concentration (blue), and outdoor-to-indoor temperature gradient (red)

What could potentially impact radon variations even more than internal temperature, is the gradient between indoor and outdoor temperatures, as it determines the pressure differences that drive airflow when the outside temperature is even lower. Figure 13 shows a comparison of the graphs of 30-day rolling mean of radon concentration and outdoor-to-indoor temperature difference based on data provided by the weather forecast website 3bmeteo (https://www.3bmeteo.com). The time series of air temperature were collected by two weather stations located near the studied catacombs: the reference station is Rome-Appio Latino for S. Callisto and Pretestato catacombs (which are less than 500 m apart) and Rome-Monteverde for the Ponziano catacomb. At S. Callisto, the temperature gradient between the outside and inside of the catacombs is almost always positive. This means that the temperature inside the catacombs remains consistently lower than the temperature outside (check Fig. 7), this is due to the proximity of the stations to one of the entrances, and to the daily opening of the catacombs to the public. This also affects the average radon levels observed, which are lower than those that could be measured if the site were undisturbed. In fact, at the catacomb of Pretestato, which has a similar geological setting but is closed to the public, the concentrations are significantly higher.

Despite the most obvious effect on radon time series measured during multiple years is the long-term fluctuation connected to the seasonal variations of temperature, weather conditions significantly influence indoor radon levels in many ways, and several meteorological parameters can affect how radon moves from the ground into enclosed spaces. Changes in atmospheric pressure are important because low-pressure systems increase radon infiltration, while high pressure suppresses it. Rainfall initially drives radon toward the surface, increasing its emanation, but heavy, prolonged rain or, anyway, high humidity, can temporarily seal soil pathways causing underground buildup that may later seep indoors. Wind effects are twofold; strong winds may draw radon indoors through cracks, but also promote ventilation, lowering concentrations.

Since the meteorological influence seems to be highly significant also in configurations that apparently should be unaffected by external ambient, such as deep boreholes, isolated tunnels (Zafrir et al., 2013) and catacombs, the relationship between the time series of radon and of these atmospheric factors, collected via the weather forecast website 3bmeteo (along with the data of indoor temperature and relative humidity recorded by the AER instruments themselves) has to be investigated.

The meteorological variables are outdoor air temperature (°C), relative humidity (%), barometric pressure (mbar), wind speed (km/s) and rainfall (mm). A plot of all data, normalised to their maximum value, and referring to station CALL1 are given as an example in supplementary Fig. 1, showing an apparently good connection between radon and temperature, and between radon and external humidity (with opposite sign, though). This is because humidity cannot be considered a completely independent variable, since its value rests on that of temperature and on the amount of water per cubic meter in the air.

**Fig. 14:**
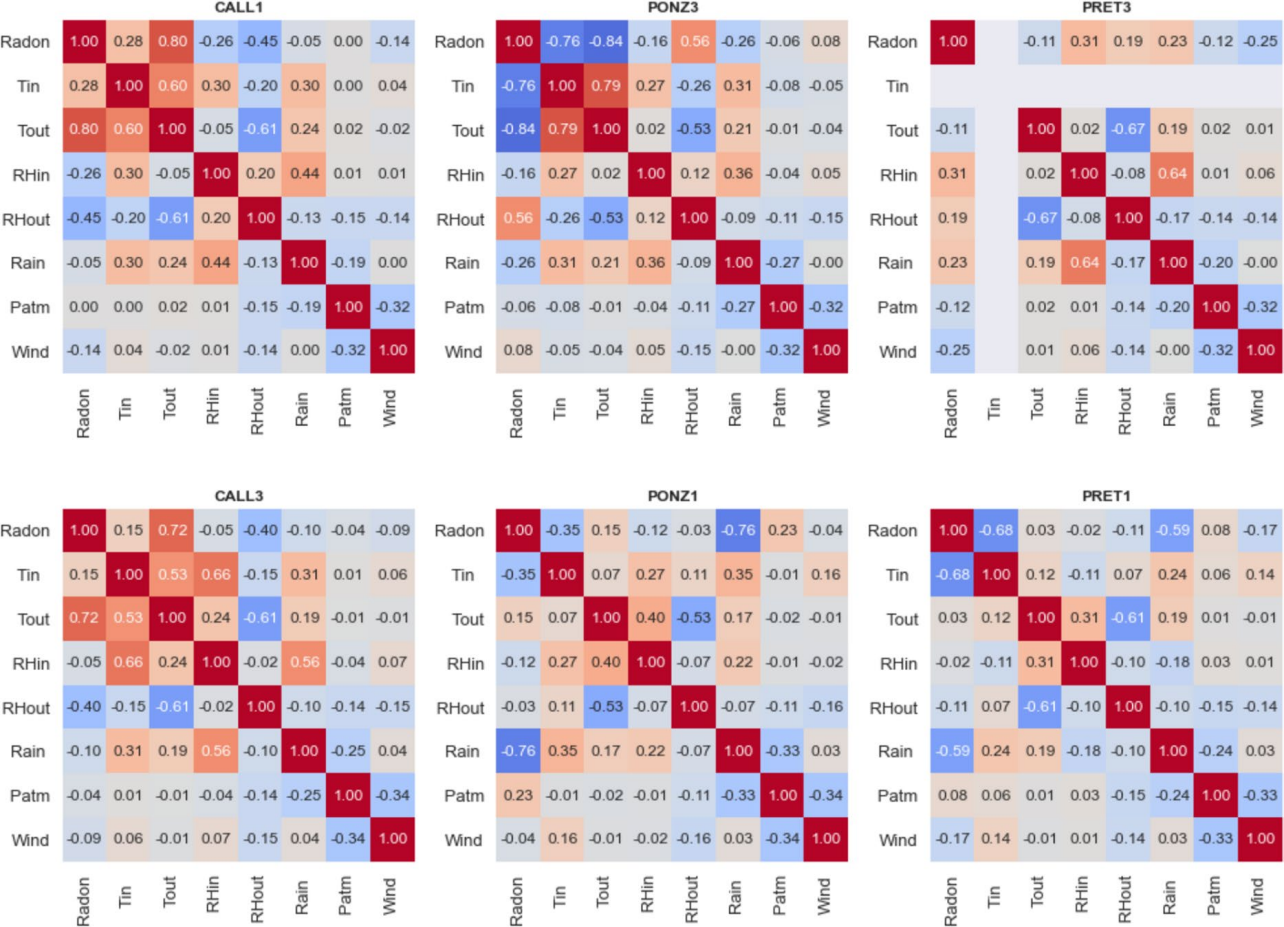
Spearman correlation matrix between radon concentration and meteorological parameters for the stations with Gore-Tex caps (top panels) and enclosed in sealed boxes (bottom panels)

For a more comprehensive analysis and a better representation of the data relationship, the Spearman correlation matrix was computed for the different stations (Fig. 14). The two stations in S.Callisto catacomb show high positive correlation between radon concentration and air temperature, both inside and outside the catacomb, and anti-correlation with outdoor relative humidity, confirming the visual indication from the previous figure. Station PONZ3 behaves in the opposite way, with radon negatively correlated with both internal and external temperatures, and positively correlated with external humidity. In the same catacomb, radon measured by station PONZ1 shows a significant correlation only with rainfall. Finally, in the Pretestato site, radon at PRET1 correlates negatively with both internal temperature and rainfall, while at station PRET3 radon does not show any relationship with the monitored meteorological parameters. All the values of correlation mentioned above were tested versus the corresponding p-values, which are all well below 0.05, proving that the relations are statistically significant.

The takeaway message is that significant correlations are often observed between radon concentration and one or more atmospheric parameters (most frequently temperature, humidity, or precipitation), but, as observed in literature (Siino et al., 2019; Soldati et al., 2020), the relative influence of each parameter is strongly site-dependent. For this reason, monitoring radon levels along with the highest number of meteorological variables is advisable and necessary to consider all possible causes underlying the temporal fluctuations of the gas.

### Implication for radioprotection

Absolute radon concentration values such as those measured during this experiment in the catacombs, which are also a workplace for archaeologists and museum staff pose serious problems in terms of workers healthcare and safety protection. As seen in the previous section, 16,754 Bq/m^3^, 2157 Bq/m^3^, and 45,418 Bq/m^3^ can be conservatively considered as a lower limit of the average level of radon concentration exposure in S. Callisto, Ponziano and Pretestato respectively, together with the evidence that seasonality effects could lead to weeks- or months-long periods where those figures are significantly exceeded. In 2020 (D.L 101 2020/07/31) and 2022 (D.L. 203 2022/11/25) Italy has implemented in its internal regulations the Euratom directive 2013/59 (European Commission, 2013) recommending a concentration limit of 300 Bq/m^3^ (in terms of the annual average concentration of radon activity in the air) as maximum permitted continuous exposure either in workplaces or private dwellings. In the presence of actual concentrations like those detected in this experiment, a severe cut of underground working time together with the implementation of active ventilation strategies, where they are possible, is mandatory. Indeed, identifying when and where radon levels surge helps in mitigating exposure risks, especially in workplaces like catacombs, where indoor air quality monitoring is key to tailoring mitigation strategies, to daily or seasonal patterns. To grant safety of the working people and environment, the cited regulatory framework prescribes specific monitoring of all the working places exposed to radon risks. As a matter of fact, the catacomb environment represents an urban but highly unusual working place, where flatly implementing monitoring procedures and protocols generically designed for common urban underground working places (basement workshops, storage facilities, sewer networks etc.) could lead to inaccurate results. Indeed, the awareness and close weighing of the typical radon concentration levels that could be found in catacombs could foster the design and proper implementation of suitable monitoring protocols.

## Conclusions

For the first time, active monitoring of three Roman catacombs was carried out, addressing the challenge of measuring indoor radon under critical conditions characterized by high gas levels combined with the effects of high humidity and temperature. After an initial test phase to organize instrument protection using protection boxes with different configurations, six time series (two for each catacomb) of at least 1.5 years in length were obtained, with a temporal resolution of 1 h. This allowed to identify periodic patterns in radon levels across different time scales.

On a seasonal scale, radon levels were observed to be higher in summer at all stations (ranging from 1 to 6 times the winter value), except for station PONZ3, located near the catacomb entrance, slightly below ground level. Higher radon levels in the warm season are an expected outcome for catacombs (as well as for caves and cellars), because they maintain a constant temperature throughout the year. This creates a density gradient that drives colder air into the catacombs during the winter, diluting the indoor radon concentration. Over shorter time scales, stations enclosed in boxes with Gore-Tex caps accurately capture small variations in gas dynamics, revealing that radon exhibits well-defined changes depending on the time of day. In contrast, sealed boxes act as low-pass filters, obscuring small-scale gas fluctuations. As for the long-term (non-periodic) trends in gas concentration variations, the analysis of the 1.5–2 year-long time series reveals a decline in radon levels in 2024 compared to 2023, coinciding with rising temperature and humidity, and decreasing gradient of temperature.

The Spearman correlation matrices show site-dependent relationships between radon and meteorological variables. In S. Callisto, radon correlates positively with temperature and negatively with humidity, while PONZ3 shows the opposite trend. Other stations exhibit varying correlations with rainfall. These findings highlight the need for comprehensive meteorological monitoring alongside radon measurements, and the awareness that the impact of environmental factors on the gas is not consistent across cases but largely depends on the characteristics of the site.

Our experiment monitored radon gas in a unique underground setting, characterized by isolation, limited accessibility, and cultural heritage significance. Active monitoring is essential to track temporal variations in radon levels across different timescales and to assess the influence of meteorological factors. Understanding these dynamics is crucial for protecting the health of visitors and, even more so, workers frequently exposed to radon. Additionally, this study serves as a valuable reference for preserving the site’s artistic and historical integrity, ensuring a safer environment while contributing to cultural heritage conservation efforts.

## Supplementary Information

Below is the link to the electronic supplementary material.

Supplementary file1 (DOCX 8297 KB)

## Data Availability

The dataset analyzed in the current study is available from the corresponding author on reasonable request.
