# Supplementary material for: Monitoring radon concentration in roman catacombs: a long-term analysis across different geological settings
Source: Environ Geochem Health. 2025 Jul 25;47(8):335. doi: 10.1007/s10653-025-02661-z (PMC12296779; doi:10.1007/s10653-025-02661-z)
Supplement: Supplementary file 1 — Supplementary file1 (DOCX 8297 KB) [file 10653_2025_2661_MOESM1_ESM.docx]

# **Supplementary Material**

| **Measurement Date** | **Radon Concentration (Bq/l)** |
| --- | --- |
| 10/03/23 | 0.7 ± 0.7 |
| 20/06/23 | 0.8 ± 0.8 |
| 12/12/23 | 1.9 ± 0.9 |
| 25/06/24 | 0.9 ± 0.9 |

Table S1 Analysis of dissolved radon concentration in water at Ponziano catacomb


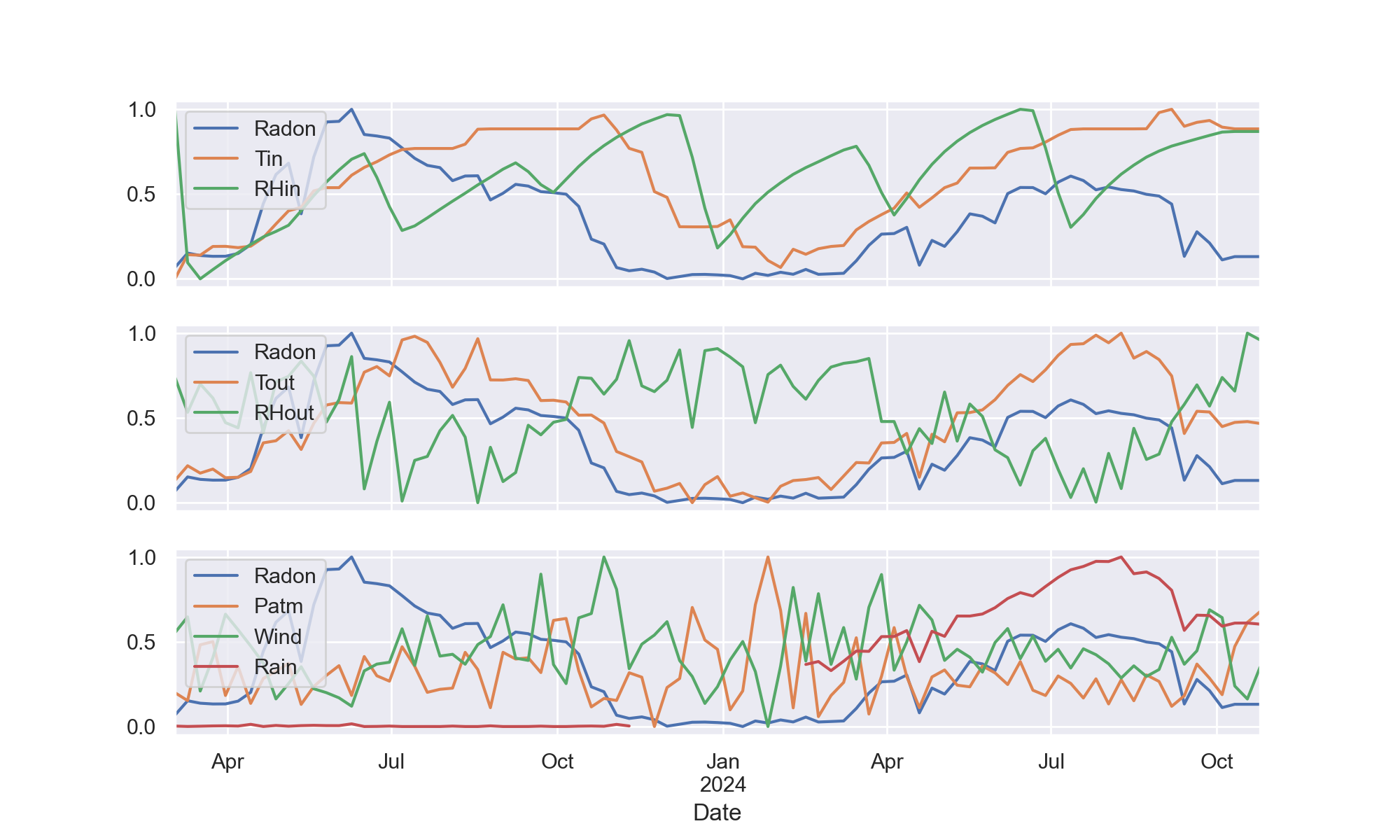
Fig S1 Weekly averages of normalised time series of radon concentration, indoor and outdoor environmental parameters relative to station CALL1
